# Immunomodulatory Effects of a Herbo-Mineral Ayurvedic Formulation in Experimental Models

**DOI:** 10.7759/cureus.58913

**Published:** 2024-04-24

**Authors:** Vaidya B Prakash, Sneha T Sati, Yashwant K Rao, Shikha Prakash, Neha Negi

**Affiliations:** 1 Immunology, Vaidya Chandra Prakash Cancer (VCPC) Research Foundation, Rudrapur, IND; 2 Clinical Research, Vaidya Chandra Prakash Cancer (VCPC) Research Foundation, Rudrapur, IND; 3 Pediatrics, Ganesh Shankar Vidyarthi Memorial Medical College, Kanpur, IND; 4 Medicine, Padaav - Specialty Ayurvedic Treatment Centre, Rudrapur, IND; 5 Clinical Research, Padaav - Specialty Ayurvedic Treatment Centre, Rudrapur, IND

**Keywords:** immbo, immunity, immuno-modulator, allergy, ayurveda

## Abstract

Background: Ayurveda, an ancient Indian system of medicine, encapsulates comprehensive principles and formulations for disease prevention and treatment. A herbo-mineral Ayurvedic formulation, IMMBO, comprising *Mandoor Bhasma *and 18 herbs has shown promising results in treating allergic rhinitis in clinical studies.

Objective: This discussed series of experimental studies were conducted to explore the immuno-modulatory potential of IMMBO.

Methodology: A series of experimental studies were carried out in immunosuppressed rats to explore the immune-modulatory effects of IMMBO.

Results: IMMBO was effective in reinstating neutrophil activation, stimulating cellular and humoral immunity, and counteracting immunosuppression at the molecular level. The modulation of key signalling molecules, including tumour necrosis factor-alpha (TNF-α), interferon-gamma (IFN-γ), interleukin-1 beta (IL-1β), extracellular signal-regulated kinase (ERK), phosphoinositide 3-kinase (PI3K), and nuclear factor-kappa B (NF-κb), showcased the formulation’s multifaceted impact. Additionally, its ability to block histamine release suggests potential in controlling allergic states, positioning it as a promising therapeutic candidate for immune-related disorders. However, the precise mode of action remains elusive, warranting further in-depth pharmacological studies.

Conclusion: This research substantiates the ancient Ayurvedic wisdom using modern scientific parameters, endorsing IMMBO's potential as an immune-modulatory agent.

## Introduction

Ayurveda, an ancient Indian system of medicine documented primarily in Sanskrit, offers comprehensive definitions, principles, and procedures for disease prevention and treatment [1,2]. Its core components include dietary practices, lifestyle recommendations, and medicinal formulations (*aahar, vihaar* and *aushadh*) [3]. Rooted in centuries-old tradition, this knowledge has been nurtured in *Gurukuls* and *Ashrams* through the revered *Guru-Shishya *tradition [4]. Ayurvedic formulations, incorporating ingredients from plant, animal, and mineral origins, have gained global recognition for their healing potential [5]. Consequently, there has been a surge in efforts worldwide to subject Ayurvedic concepts, principles, and formulations to rigorous research methodologies.

The Herbo-Mineral Ayurvedic Formulation (HMAF) under study in the present study is a combination of 18 herbs and *Mandoor Bhasma*, was initially pioneered by a registered Ayurvedic physician in North India, operating under the provisions of Section 33EEC of the Drug and Cosmetics Act of India [6]. The formulation has shown promising results in the treatment of patients suffering from persistent and intermittent forms of allergic rhinitis. Following successful outcomes, a manufacturing license was obtained from the State Drug Licensing Authority in India for its commercial production, and it was introduced into the market under the name IMMBO (Manufacturing License No. A-3077/2000).

Simultaneously, IMMBO underwent a phase III prospective randomized controlled clinical trial to evaluate its efficacy in comparison to the standard treatment of fixed-dose combination (FDC) levocetirizine 2.5 mg + montelukast 4 mg in allergic rhinitis patients. The study yielded compelling evidence supporting IMMBO's effectiveness in treating allergic rhinitis [7].

Numerous studies have explored the connections between Ayurveda and Immunology, aiming to bridge ancient wisdom with modern scientific understanding. Ayurvedic texts describe the concept of "*Ojas*," believed to represent the body's vital energy and immune strength, underscoring the importance of immune balance in maintaining health [8]. Modern research has corroborated these claims, demonstrating the immunomodulatory effects of various Ayurvedic herbs and formulations. For example, studies have highlighted the immune-enhancing properties of herbs such as *Ashwagandha (Withania somnifera), Guduchi (Tinospora cordifolia)*, and *Amalaki (Emblica officinalis)*, which exhibit antioxidant, anti-inflammatory, and immunostimulatory activities [9,10]. Furthermore, Ayurvedic formulations like *Chyawanprash *and *Triphala* have been shown to modulate immune responses by regulating cytokine production, enhancing phagocytic activity, and promoting T-cell proliferation [11].

Based on its clinical efficacy and available literature, IMMBO underwent extensive *in vivo *and *in vitro* studies to explore its possible mechanism of action. Wistar albino rats were subjected to experiments involving cyclophosphamide (CPH)-induced immune suppression [12]. These studies assessed IMMBO's impact on various parameters, including neutrophil adhesion, delayed-type hypersensitivity response, antibody levels, inflammatory and pro-inflammatory markers, and cell signalling pathways. This paper delves into the outcomes and interpretations derived from these studies. A preprint of this article was previously posted on Research Square on January 26, 2024.

## Materials and methods

The test material used was IMMBO (Table 1).

**Table 1:** Composition of IMMBO * Prepared using in-process control grinding at particle size <2 µm and heated up to 770° C in a programmed muffle furnace (*Gaja Puta*).

Ingredients	Quantity (per 1000 mg)
Cedrus deodara, Curcuma longa, Cypus rotundus, Emblica officinalis, Emblica ribes, Holarrhena antidysentrica, Picrorrhiza kurroa, Berberis aristata, Piper longum, Piper longum (Root), Piper nigrum, Plumbago zeylanica, Saussurea lappa, Terminallia belerica, Terminallia chebula, Zingiber officinalis	25 mg each
Boerhavia diffusa, Operculina turpethum	50 mg each
Mandoor Bhasma*	500 mg

Animals of the same sex, housed in groups of two to three per cage, were kept in a controlled environment within an air-conditioned room with a temperature maintained at 20 ± 3 °C and a relative humidity ranging from 30% to 70%. The room had air changes occurring at a rate of 10-15 per hour, and a 12-hour light and 12-hour dark cycle was maintained. Temperature and humidity levels were continuously monitored and recorded once daily.

The standard polycarbonate cages were equipped with stainless steel mesh top grills and provisions for pelleted food and water bottles. All the animals underwent a minimum five-day acclimatization period in a laboratory setting. Veterinary examinations were conducted on the day the animals were received and before the commencement of the treatment.

Animals were fed with normal rodent feed and reverse osmosis water was always available to the animals during the experiments. Daily observations for any clinical signs were conducted.

Study design

The study was conducted in South India by an internationally accredited Department of Scientific and Industrial Research (DSIR), a Government of India-recognized research unit. Healthy Wistar albino rats (six to eight weeks old) were grouped based on body weight and randomly assigned to different treatment groups. In the main study, 56 rats were divided into seven experimental groups (G1 to G7) for immune modulator study. Additionally, a pilot set of rats (G8 to G10; each with n=8) was maintained separately to evaluate delayed-type hypersensitivity (DTH).

Ethics

This study was performed at a CPCSEA-approved and AAALAC accredited laboratory following all ethical practices as laid down in the guideline/s for animal care. The study was approved by the Institutional Animals Ethics Committee (IAEC) of the test facility vide ethical clearance number VIP/IAEC/324/2021.

Materials

CPH served as the negative control, functioning as an immunosuppressant, while levamisole hydrochloride acted as the positive control, serving as an immunity stimulant [12,13].

Fresh sheep blood was collected and mixed with sterile Alsever's solution. The mixture was centrifuged at 3000 rpm for five minutes to obtain red blood cell sediment. The cell pellet was then resuspended in phosphate buffer saline (PBS) with a pH of 7.2 and centrifuged at 1500 rpm. This washing process was repeated until a clear top layer was observed. The sheep red blood cells (SRBC) were adjusted to the desired levels based on haematocrit values and stored in the refrigerator for later use.

RAW 264.7 macrophages and RBL-2H3 mast cells were obtained from American Type Culture Collection (ATCC), USA and maintained in Dulbecco's Modified Eagle Medium (DMEM) with 5% fetal bovine serum (FBS)/1% penicillin-streptomycin at 37°C in a 5% CO_2_ environment. Both cell types were incubated in DMEM with 10% FBS, 2 mM glutamine, 100 U/mL penicillin, and 50 μg/mL streptomycin.

Experimental protocol

In set 1, rats in the normal control group (G1) received saline for two weeks. Experimental groups (G2 to G7) received CPH (100 mg/kg) on Day 0, followed by the SRBCs challenge on Days 7 and 14. G4 received the immunostimulant levamisole (20 mpk). G5 to G7 received IMMBO at low (200 mpk), mid (400 mpk), and high (800 mpk) doses orally (Table 2).

**Table 2:** Workflow SRBC: sheep red blood cells; DTH: delayed-type hypersensitivity; TNF-α: tumour necrosis factor-alpha; IFN-γ: interferon-gamma; IL-6: interleukin-6; IL-1β: interleukin-1 beta; IL-10: interleukin-10; IgG: immunoglobulin G; IgA: immunoglobulin A; IgM: immunoglobulin M; AKT: protein kinase B; ERK: extracellular signal-regulated kinase; PI3K: phosphoinositide 3-kinase

Test groups	Endpoint assessments			
	Immune suppression	SRBC (1-2%)	Treatment	Day 15
	Day 0	Day 7 & 14	Day 1 to 14	
G1	-	-	Vehicle (0.9% saline)	Body and lymphoid organs weight, Neutrophil adhesion, DTH, TNF-α, IFN-γ, IL-6, IL-1β, IL-10, IgG, IgA, IgM, MAP kinases (AKT, ERK, PI3K)
G2	Cyclophosphamide (100mpk)	-	-	
G3		SRBC (Control, IP)	-	
G4		SRBC (1-2%, IP)	Levamisole (20 mpk, po)	
G5		SRBC (1-2%, IP)	IMMBO (200 mpk, po)	
G6		SRBC (1-2%, IP)	IMMBO (400 mpk, po)	
G7		SRBC (1-2%, IP)	IMMBO (800 mpk, po)	
G8		SRBC footpad control for DTH	-	DTH
G9		SRBC footpad	Levamisole (20 mpk, po)	
G10		SRBC footpad	IMMBO (800 mpk, po)	

In set 2, animals in G8 to G10 were subjected to DTH testing with SRBC on Days 7 and 14. G9 received levamisole (20 mpk) and G10 received high-dose IMMBO (800 mpk) from Day 1 to Day 14 as pre-treatment (Table 2).

In vivo assessments

Neutrophil Adhesion

On Day 14 after administering the test item, blood samples were taken from rats in Groups 5, 6, and 7. These samples were initially analyzed for total leukocyte counts (TLC) and differential leukocyte counts (DLC). Afterwards, they were incubated with 80 mg/mL nylon fibres for 15 minutes at 37°C.

The product of TLC and the percentage of neutrophils were given as the neutrophil index (NI) of the blood sample. Per cent neutrophil adhesion was calculated as follows:

$\text{Neutrophil adhesion} = \frac{(NIu - Nlt) \times 100}{NIu}$

where NI untreated (NIu) is the neutrophil index of the untreated blood sample; NI treated (NIt) is the neutrophil index of the treated blood sample.

Determination of Delayed-Type Hypersensitivity Responses

On Day 7 of the study, animals in groups G8 to G10 received an injection of 0.1 mL containing 1x 10^8^ SRBC into the right hind footpad, while the left footpad received 0.1% PBS as a control. Test group G9 received daily treatment with IMMBO at 800 mpk from Days 1 to 14, and reference group G10 received levamisole at 20 mpk following the same schedule. On the 14th day, all animals were challenged with another subcutaneous injection of 0.1 mL containing 1 x 10^8^ SRBCs into the left hind footpad. To assess DTH, footpad thickness was measured using a Vernier calliper on both the right and left hind paws by using the formula:

$\frac{\text{Left footpad challenged with antigen} - \text{Right footpad control}}{\text{Left footpad challenged with antigen}} \times 100$

Determination of Pro-inflammatory Factors and Total Antibody Levels

Major inflammatory cytokines namely nuclear factor-kappa b (NF-κB), tumour necrosis factor-alpha (TNF-α), interferon-gamma (IFN-γ), interleukin-6 (IL-6), interleukin-1 beta (IL-1β), interleukin-10 (IL-10), inducible nitric oxide synthase (iNOS) (in spleen tissue) were assessed in specified matrices. The three major classes of immunoglobulins (IgG, IgA, and IgM) levels were assessed in serum.

Western Blotting for Cell Signaling Markers/Mitogen-Activated Protein (MAP) Kinases 

Western blot experiments were conducted to assess MAP kinase expression levels, including protein kinase B (AKT), extracellular signal-regulated kinase (ERK), and phosphoinositide 3-kinase (PI3K), using spleen tissue lysed with Radio-Immunoprecipitation Assay Buffer (RIPA) Buffer. The procedure involved:

Tissue collection: Spleen tissues were weighed, snap-frozen in liquid nitrogen, and homogenized in ice-cold RIPA Buffer containing protease and phosphatase inhibitors.

Protein extraction: Samples were centrifuged, and the supernatant was collected for analysis. Protein content was determined using the Bradford method with bovine serum albumin (BSA) as the standard.

Sodium dodecyl-sulfate polyacrylamide gel electrophoresis (SDS-PAGE): Proteins were separated on 12.5% SDS-PAGE gels, with pre-stained markers included for reference.

Membrane transfer: Proteins were transferred to polyvinylidene fluoride (PVDF) membranes using the wet blot method.

Antibody incubation: The membrane was blocked with 5% skimmed milk in tris-buffered saline with Tween-20 (TBST) and then incubated with primary antibodies overnight at 4°C. After washing, secondary antibodies were applied for one hour at room temperature.

Washing: Blots were washed three times with TBST.

Detection: ALP-conjugated antibodies were used for colour development, and blot images were captured. Band densities were quantified using ImageJ software (National Institutes of Health, Bethesda, USA).

Normalization: Band densities were normalized to the glyceraldehyde 3-phosphate dehydrogenase (GAPDH) signal, serving as the loading control.

In vitro assessments

Stimulation of RAW Cells With Lipopolysaccharide (LPS) and Enzyme-Linked Immunosorbent Assay (ELISA) for Cytokines

In this experiment, RAW 264.7 cells were seeded at 0.3 x 106 cells/mL in six-well plates and treated with varying concentrations of IMMBO. The cells were also exposed to the inflammatory stimulant LPS at 1 µg/mL for 24 hours. After treatment, cell lysates were collected and analyzed to measure NF-κB and TLR-4 levels. NF-κB regulates immune and inflammatory genes, while TLR-4 detects LPS, a bacterial component triggering immune responses. This analysis assesses how IMMBO and LPS interact to affect NF-κB pathway activation and TLR-4 expression, providing insights into the potential anti-inflammatory or immunomodulatory effects of IMMBO in response to LPS stimulation.

*Assessment of IMMBO* *on RBL-2H3 Cells Degranulation*

In this experiment, degranulation in RBL-2H3 cells was assessed by measuring β-hexosaminidase release. The process involved the following steps:

Sensitization: RBL-2H3 cells were plated (1x10^5 cells/well in a 24-well plate) and sensitized with 0.2 μg/mL monoclonal anti-dinitrophenyl mouse Immunoglobulin-E (DNP-IgE) overnight at 37°C.

Washing: The cells were washed with PIPES (piperazine-N,N'-bis(2-ethanesulfonic acid)) buffer to remove DNP-IgE.

Test item treatment: The cells were incubated with different test item concentrations for 30 minutes at 37°C.

Degranulation induction: The cells were treated with 1 μg/mL human DNP-albumin and further incubated for 30 minutes at 37°C to induce degranulation.

β-hexosaminidase release: After degranulation, 25 μL of cell supernatant was transferred to a 96-well plate containing 25 μL of 5 mM 4-nitrophenyl N-acetyl-β-D-glucosaminide in 0.1 M citrate buffer (pH 4.5) and incubated for two hours.

Reaction termination: The reaction was stopped by adding 200 μL of a stop buffer (0.05 M Na2CO3/0.05 M NaHCO3, pH 10).

Measurement: The optical density was measured at 405 nm to assess degranulation.

Statistical analysis

The data was entered in the Microsoft Excel spreadsheet (Microsoft® Corp., Redmond, WA), where it was organized and then exported to the statistical software GraphPad Prism (Version 9.0, GraphPad Software, San Diego, CA) for further statistical analyses. It was observed that the data conformed to the assumptions of parametric data. One-way analysis of variance (ANOVA) was used to test the significance between means of respective control vs treatment/exercise groups. The data was further subjected to Dunnett’s test for multiple comparisons. All results have been expressed as the mean ± standard deviation (SD) and presented in tables and/or graphs. All differences were considered statistically significant if p ≤ 0.05. The GraphPad Prism software performed all statistical analyses.

## Results

Determination of neutrophil adhesion

CPH treatment completely suppressed neutrophil activation compared to untreated controls (G1 versus G2). However, treatment with the immune-stimulant reference compound levamisole restored neutrophil activation (G1 versus G4). IMMBO was effective at mid- and high-dose levels in reinstating neutrophil activation to normal levels (G1, G6, and G7), but it had no significant effect at low doses (G5). IMMBO appears to be an effective method for activating the immune system in immune-suppressed animals (Table 3).

**Table 3:** Effect of IMMBO on neutrophil adhesion test in rats CPH: cyclophosphamide; SRBC: sheep red blood cells

Groups	Treatment	% of neutrophils adhered to nylon
G1	Native control	6.06
G2	CPH alone	-11.36
G4	CPH + SRBC + Levamisole	5.19
G5	CPH + SRBC + IMMBO 200mpk	1.95
G6	CPH +SRBC + IMMBO 400mpk	5.19
G7	CPH + SRBC + IMMBO 800mpk	6.06

Determination of DTH responses

**Figure 1:**
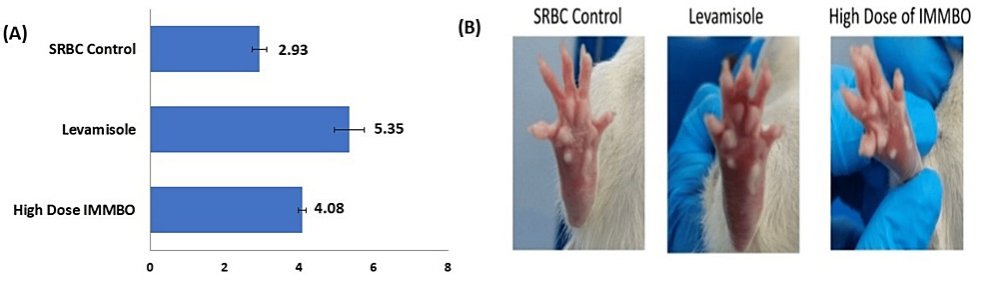
Outcome of the delayed type hypersensitivity study The graph in Figure 1A denotes mean paw thickness (in mm) in the three study groups; Figure 1B shows increased paw thickness in the animals. SRBC: sheep red blood cells

The foot pad swelling assay in rats is used to assess DTH responses. In this model, rats' foot pads are sensitized with an antigen (SRBC), and after seven days a second dose of the antigen is given. The tissue responds in the form of swelling. Results show that CPH and IMMBO at 800mpk dose is effective in stimulating cellular immunity in an immune-suppressed condition (Figures 1A-1B).

Estimation of antibody and inflammatory biomarkers in blood and serum

The effect of the IMMBO on humoral immune response was assessed by measuring levels of IgG, IgM, and IgA antibodies in CPH-induced immunosuppressed rats. The quantification of the antibodies was done by the ELISA kit method. Results are presented in Table 4. CPH treatment caused a rapid decline in the content of all three antibody classes, and treatment with levamisole overcame this effect. Levamisole was found to increase circulating antibodies to significantly higher levels compared to untreated control animals. Treatment with IMMBO was found to be effective at all three dose levels, and for IgM and IgG, it compensated more than the control levels. The effect of IMMBO was similar to that achieved with levamisole. This data demonstrates that IMMBO is a powerful stimulator of humoral response and has the potential for use in immune-suppressed states such as chemotherapy.

**Table 4:** Effect of IMMBO on IgM, IgG, and IgA levels in sera in response to SRBC in cyclophosphamide-induced Wister rat. Mean ± SD (n = 8). The level of significance was adjusted using four groups (CPH + SRBC + Levamisole/low-dose IMMBO/mid-dose IMMBO/high-dose IMMBO). CPH: cyclophosphamide; SRBC: sheep red blood cells

Groups	IgM (ng/mL)	IgG (ng/mL)	IgA (ng/mL)	p-value
Control group	247±12.3	320.74±32.4	219.89±33.2	
CPH alone	172.22±10.1	114.83±21	168.46±15.8	
CPH + SRBC + Levamisole	455.29±39.3	496.83±38.3	659.87±45.1	
CPH + SRBC + Low-dose IMMBO	437.81±22.5	379.1±56.3	215.7±68.7	p-value < 0.0001
CPH + SRBC + Mid-dose IMMBO	441.37±32.1	314.53±88	361.01±47.2	p-value < 0.0001
CPH + SRBC + High-dose IMMBO	460.26±21.7	341.38±91.8	502.82±29.5	p-value 0.00002

Pro-inflammatory cytokine levels after IMMBO treatment of immune-suppressed animals

**Figure 2:**
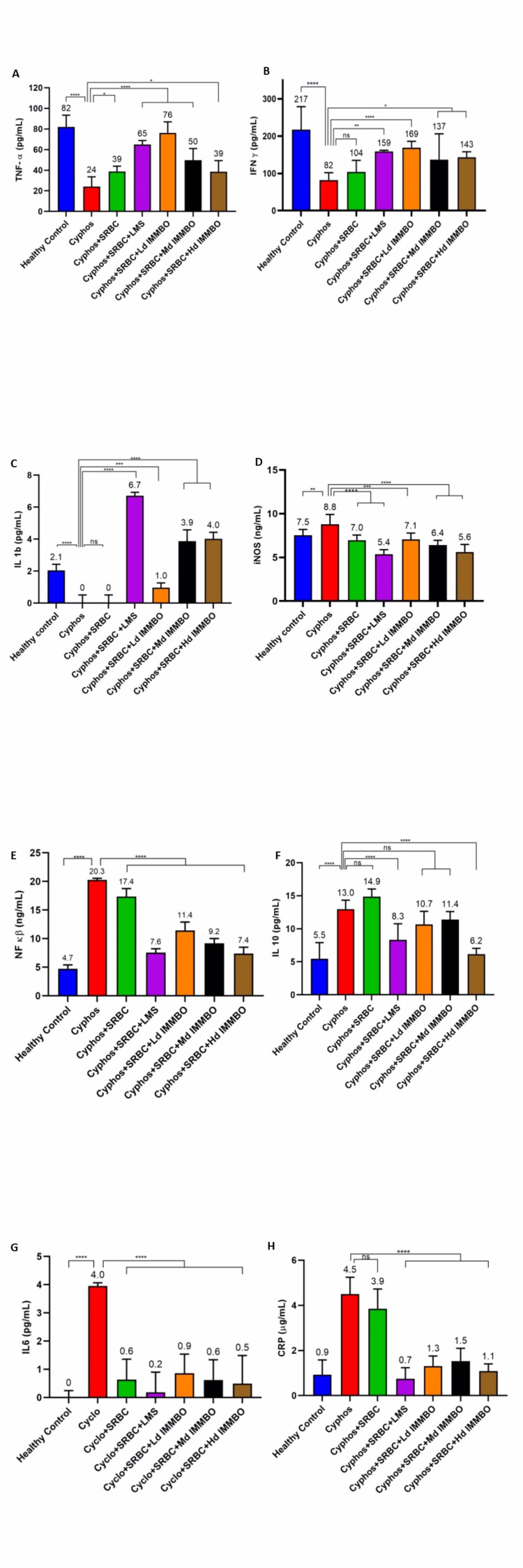
Modulation of cytokine expression in the spleen of immune-suppressed rat after treatment with IMMBO. Rats (n=8/group) were rendered immune-suppressed by treatment with cyclophosphamide and stimulated with SRBC as an external antigen. Immune modulatory effect of IMMBO was tested at three different dose levels after repeated oral dosing for 10 days. The spleen was collected from the animals, and the homogenates were assayed for expression of various biomarkers by specific ELISA kits. Statistical analysis was performed using GraphPad prism. Statistical differences between groups were evaluated using a one-way analysis of variance followed by Dunnett's test or t-test. P<0.05 was considered to indicate a statistical significance (where **** refers to p≤0.0001, *** refers to p≤0.001, ** refers to p≤0.01 and * refers to p≤0.05). SRBC: sheep red blood cells; ELISA: Enzyme-Linked Immunosorbent Assay; TNF-α: tumour necrosis factor-alpha; IL-1β: interleukin-1 beta; NF-κb: nuclear factor-kappa B; IL6: interleukin-6; IFN-γ: interferon-gamma; iNOS: inducible nitric oxide synthase; IL10: interleukin-10; CRP: C-reactive protein

To evaluate the protective effect of IMMBO on immunosuppression in CPH-treated rats, IMMBO was orally administrated for 10 days at three different dose levels. The levels of cytokines and immunoglobulin in the serum and spleen were evaluated by ELISA. Immunomodulating cytokines namely NF-κB, TNF-α, IFN-γ, IL-6, IL-1β, IL-10, and iNOS in spleen tissue homogenate assessed as markers for effectiveness. Results are summarized in Figure 2. In comparison with the untreated control, the group treated with CPH alone showed a significant reduction in the expression of TNF-α, IFN-Ƴ, and IL-1β. (~2.6, 3.4, and 2.1 folds respectively compared to untreated animals). Levamisole corrected the expression levels to almost similar levels seen in untreated control animals. IMMBO treatment also rescued the cytokines expression levels, but a clear dose response was not seen. In the case of TNF-α and IFN-ϒ, the rescue was already high at a low dose of IMMBO (3.1-fold and 6.7-fold, respectively), and a further increase in dose level did not improve the rescue effect. Expression of iNOS was not affected by CPH treatment (Panel D, Figure 2). Levamisole or IMMBO treatment did not change the level of expression compared to untreated control animals. NFkB is a transcription factor, and controls expression of various genes coding both anti- and pro-inflammatory cytokines. Treatment with CPH caused a significant increase in expression of NF-kb and interestingly levamisole treatment reduced the expression level to almost reaching that of control untreated naïve animals (Panel E, Figure 2). Treatment with IMMBO also reduced its expression and a similar level of reduction was seen at the three dose levels used. Interestingly expression levels of IL-10, IL-6, and CRP were elevated in CPH-treated animals, and their levels were suppressed to normal levels when levamisole was used (Panels F, G, and H). Suppression of expression was also seen in IMMBO-treated animals, therefore demonstrating an effect similar to the reference drug levamisole. These results are thought-provoking as immunosuppression generally down-regulates inflammatory cytokines and markers. The results obtained in this study are likely due to the combined use of SRBC antigen and cyclophosphamide in all test groups, which would have impacted the immune system in an unknown manner.

Cell signalling pathways in spleen tissue after IMMBO treatment of immune-suppressed animals

**Figure 3:**
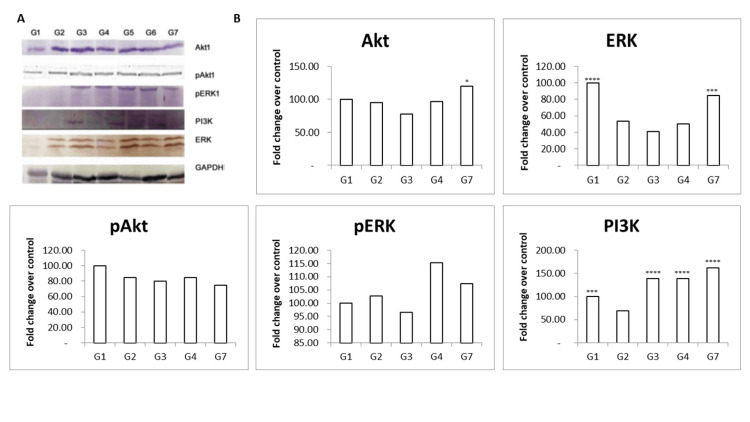
(A) Western blot analysis of spleen tissue homogenates for expression of selected signalling proteins. Proteins were separated on a 12.5% SDS-PAGE and after transfer to a nitrocellulose membrane, the blots were developed using specific primary and secondary antibodies; (B) The density of bands was measured using ImageJ software. P<0.05 was considered to indicate a statistical significance (where **** refers to p≤0.0001, *** refers to p≤0.001, ** refers to p≤0.01 and * refers to p≤0.05). SDS-PAGE: sodium dodecyl-sulfate polyacrylamide gel electrophoresis; Akt: protein kinase B; ERK: extracellular signal-regulated kinase; pAkt: phosphorylated protein kinase B; pERK: phosphorylated extracellular signal-regulated kinase; PI3K: phosphoinositide 3-kinase

The spleen is involved in a wide range of immunological functions and regulates both T and B cell responses to antigenic targets in the blood. Its role in haematopoiesis is well known. Components of MAPK and PI3K/AKT signalling pathways are well studied in spleen and immune regulation. Therefore, an attempt was made to study the effects of IMMBO treatment on the regulation of selected signal cascades in spleen tissue. The levels of MAPK (pERK1/2), PI3K, and AKT activation were determined using Western blot technique. At the end of the experimentation, animals were euthanized and the spleen was collected. Tissue was homogenized using a lysis buffer containing protease and phosphatase inhibitors. After clarification by centrifugation, the supernatants were used for the western blot. Comparable protein amounts of homogenate were loaded in each gel. The results of this experiment are shown in Figure 3. The levels of ERK and PI3K were reduced significantly after treatment with CPH (compare G1 and G2, Panel B). For ERK, the reduction was corrected to almost normal levels at the high dose of IMMBO (compare G1 and G7, Panel B). For PI3K, the correction to normal levels was seen at the lowest dose of IMMBO tested and did not improve any more at the next dose levels. We were unable to see clear changes in expression patterns for other targets such as Akt1, pAkt1, and pERK1, probably due to the lack of sensitivity of primary antibody preparations used in the study. However, the positive effect of IMMBO on key signalling molecules (ERK and PI3K) is an interesting observation. Both these proteins are known to be involved in immune modulation and the western blot data corroborates earlier data presented in this report on immune modulation with IMMBO.

Regulation of NF-κB expression by IMMBO in RAW264.7 cells

**Figure 4:**
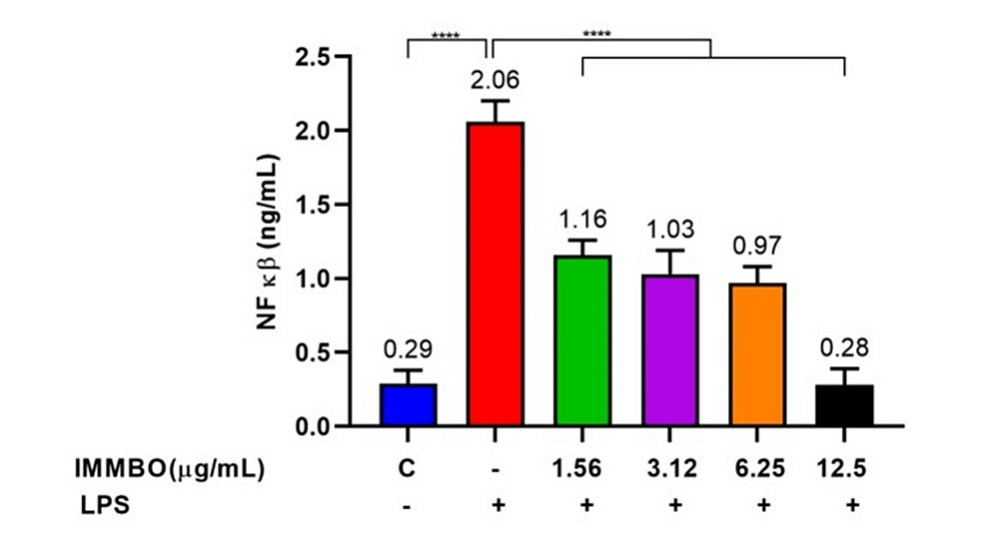
Effect of IMMBO on NF-kb in LPS-treated RAW 264.7 cells **** refers to p≤0.0001 NF-κB: nuclear factor-kappa b; LPS: lipopolysaccharide

Stimulation with *Escherichia coli* LPS triggers the expression of pro-inflammatory cytokines and biomarkers in cells. These can be measured using ELISA assays. To study the effect of IMMBO, RAW cells were seeded in six-well plates (0.3 x 10^6^ cells/mL). The cells were pre-treated with IMMBO at different concentrations, with or without LPS (1 µg/mL) for 24 h. Cell supernatant was collected by centrifugation and analyzed for the expression of NF-κB (Figure 4). The result showed that LPS significantly increased the NF-κB level (~10 folds compared to untreated control cells). IMMBO treatment resulted in a concentration-dependent inhibition of NFkb expression, and at the highest dose of IMMBO, NFkb levels were found similar to untreated cells.

Effect of IMMBO treatment on degranulation of RBL-2H3 cells

**Figure 5:**
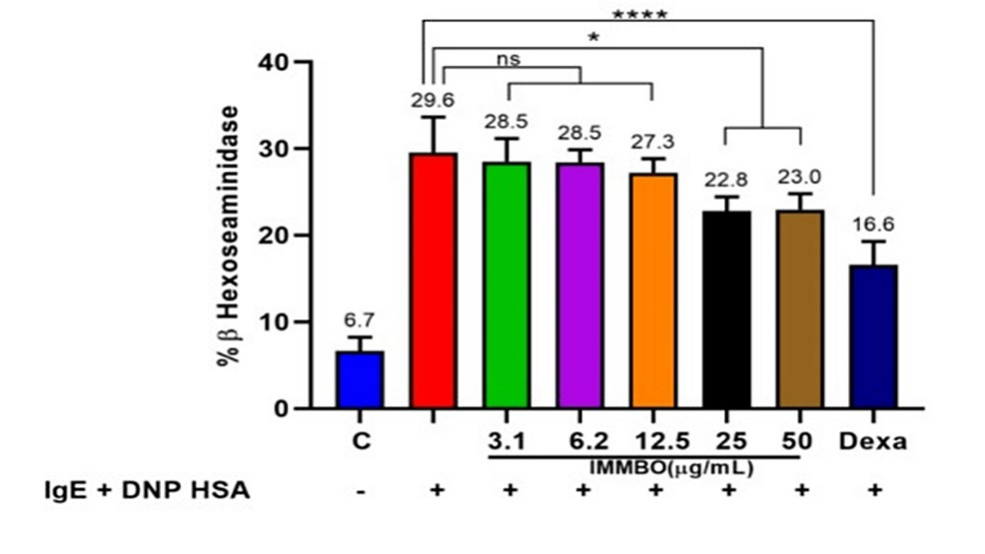
Effect of IMMBO on degranulation in stimulated RBL 2H3 cell line where **** refers to p≤0.0001, *** refers to p≤0.001 and * refers to p≤0.05. IgE: immunoglobulin E; DNP: dinitrophenol; HSA: human serum albumin

IgE-activated cells release histamine, measured with ELISA assays. These cells were used to test IMMBO's ability to block histamine release in allergies. Degranulation was detected by measuring β-hexosaminidase release in the medium. Results are shown in Figure 5. IgE-DNP human serum albumin (HSA) stimulated cells showed a ~six-fold increase in β-hexosaminidase release compared to unstimulated control cells. Control compound dexamethasone (100µM) significantly reduced the histamine level. IMMBO treatment was effective albeit at low levels, and the effect was clearly seen at higher concentrations (25ug/mL and above). Higher concentrations of IMMBO could not be tested due to challenges of media dilution, etc. that impacted assay performance. Overall, results indicate that IMMBO at higher concentrations can block histamine release and thus has the potential to be used in controlling allergic states.

## Discussion

The Ayurvedic pharmacopoeia is enriched with an extensive array of classical formulations, incorporating elements from plant, animal, and mineral sources, either individually or in combination [14]. These formulations, crafted over time by revered *Rishis* and *Munis* of ancient India, were rooted in visionary and intuitive wisdom, complemented by keen observations. The sages documented the formulas along with brief descriptions of preparation methodologies and recommended usage with doses. However, applied aspects of Ayurvedic therapy were more hands-on back then, guided by a *Guru* and the knowledge was transmitted through generations, adhering to the *Guru Shishya* Tradition of the era. Present-day Ayurveda primarily relies on these ancient descriptions, lacking modern pharmacological explanations.

IMMBO is a modified and standardized form of a classical Ayurvedic formulation called *Punarnavadi Mandoor* [15]. IMMBO was first found effective in treating patients suffering from intermittent and persistent allergic rhinitis in a North India-based Ayurvedic clinical practice. IMMBO is prepared using 18 herbs and *Mandoor Bhasma*. The herbs used in the preparation are dominant in *katu* and *tikht ras, katu vipak, ushna virya* and *ruksha* and *laghu* *guna*. These are able to eliminate *kapha* and *pitta* but may aggravate *vata*. *Mandoor Bhasma* which is present in equal quantity is predominantly *madhur*, *tikht* and *kashay ras* with *madhur vipak* and *sheet virya*. *Madhur ras* is able to suppress *vata* and aggravate *kapha*. In a nutshell, IMMBO is a *pitta kapha nashak, shothagna* (anti-inflammatory) medicine. The therapeutic efficacy of IMMBO could easily be explained following Ayurvedic concepts of *Vata, Pitta, Kapha,* and other essential parameters derived from Ayurvedic principles of diagnosis and treatment. However, it is difficult to explain it in today's scientific terminology. Hence, a proof of efficacy study was carried out on IMMBO as a phase III randomised controlled comparative clinical under subject experts of modern medicines. IMMBO depicted promising clinical efficacy in reducing IgE levels and total nasal symptom scores in the study [7].

IMMBO is a complex compound containing herbs and minerals. None of the ingredients possesses properties to treat allergic rhinitis when used alone, but act differently when used in a compound form, indicating the synergistic effect of herbs and minerals. In the above background, exploratory experimental studies were carried out on IMMBO to understand the science behind the seen effect in alleviating the symptoms of allergic rhinitis.

The findings of the experimental studies shed light on the immuno-modulatory potential of IMMBO. The observed effectiveness of IMMBO in reinstating neutrophil activation under immune-suppressed conditions is a key indicator of its immunostimulant properties. The foot pad swelling assay reveals the ability of IMMBO to stimulate cellular immunity in immune-suppressed conditions induced by CPH. The ability of IMMBO to compensate for the decline in antibody levels induced by CPH, akin to the reference drug levamisole, positions it as a potent stimulator of the humoral response. The observed reduction in ERK and PI3K levels following CPH treatment, and their subsequent restoration by IMMBO, suggests that IMMBO may act by regulating essential signaling pathways involved in immune modulation. This mechanism of action highlights the potential of IMMBO as a promising candidate for immune-related disorders, where dysregulation of these signalling pathways is commonly observed. A limitation of the present study was that other protein targets could not be targeted due to a lack of sensitive antibody preparations available for experimentation. However, the study on NF-κB expression in RAW264.7 cells adds depth to our understanding of IMMBO's anti-inflammatory properties. The inhibition of NF-κB, a crucial transcription factor involved in the expression of pro-inflammatory genes, suggests that IMMBO may exert its effects by suppressing inflammatory responses at the molecular level. The ability of IMMBO to block histamine release in IgE-activated cells suggests its potential in controlling allergic states. The results of the present study are very encouraging and advocate Ayurvedic knowledge by using modern scientific parameters and technology.

The comprehensive exploration of various immunological parameters provides valuable insights into its potential applications. These findings highlight the potential role of IMMBO as a potential therapeutic agent for conditions characterized by immunosuppression and inflammation. By targeting key cytokines and signalling pathways, IMMBO holds promise for the development of novel treatments for a range of immune-related disorders. But the exact mode of action of IMMBO remains unclear. It will be worthwhile to carry forward in-depth pharmacological studies to develop intriguing science behind the immune-enhancing characteristics of IMMBO starting its administration into the human body and its pathways from buccal mucosa to cellular metabolism.

## Conclusions

In conclusion, the results presented in this study collectively underscore the immunomodulatory potential of IMMBO, a herbo-mineral Ayurvedic formulation. The observed effectiveness of IMMBO in instating immune responses under various experimental conditions positions it as a promising candidate for further research and potential therapeutic applications in immune-related disorders.
